# Heptamethine carbocyanine DZ-1 dye for near-infrared fluorescence imaging of hepatocellular carcinoma

**DOI:** 10.18632/oncotarget.18131

**Published:** 2017-05-24

**Authors:** Jiaze An, Ningning Zhao, Caiqin Zhang, Yong Zhao, Dengxu Tan, Ya Zhao, Bing Bai, Hai Zhang, Boyang Jason Wu, Changhong Shi

**Affiliations:** ^1^ Laboratory Animal Center, The Fourth Military Medical University, Xi’an, Shaanxi 710032, China; ^2^ Department of Hepatobiliary and Pancreaticosplenic Surgery, Xijing Hospital, The Fourth Military Medical University, Xi’an, Shaanxi 710032, China; ^3^ Department of Pharmaceutical Sciences, College of Pharmacy, Washington State University, Spokane, WA 99210, USA

**Keywords:** heptamethine carbocyanine, hepatocellular carcinoma, HIF1α, near-infrared fluorescence, organic anion-transporting polypeptide

## Abstract

Near-infrared fluorescence (NIRF) dyes have recently emerged as promising tools for non-invasive imaging of different types of cancers. Here, we explored the potential utility of a NIRF DZ-1 dye, with dual imaging and tumour targeting functions, in hepatocellular carcinoma (HCC). We showed the preferential uptake of DZ-1 by HCC cells *in vitro* and in derived subcutaneous/orthotopic tumour xenografts, accompanied by a minimal effect on normal cells. DZ-1 simplified tumour growth profiling as well, since we were able to correlate NIRF signals with tumour volume and/or tumour-emitting luminescence in mice. Using both orthotopic tumour transplantation and cirrhosis models in parallel, we demonstrated the ability of DZ-1 to differentiate liver tumour from cirrhosis. DZ-1 showed superiority in HCC imaging over indocyanine green by demonstrating significantly enhanced tumour-targeting specificity. At the cellular level, DZ-1 was mainly retained in mitochondria and lysosomes. Additionally, DZ-1 fluorescence spectroscopy has been used for the intraoperative navigation of rabbit liver cancer, to determine surgical margins. We showed that tumor hypoxia and select organic anion-transporting polypeptide genes mediate NIRF dye uptake in HCC, which was supported by clinical evidence. All these findings represent the first evidence that DZ-1 is an effective molecular probe for tumour-specific imaging in HCC, and provide insights into the development of a new generation of imaging agents for intraoperative guidance of cancer surgery.

## INTRODUCTION

Near-infrared fluorescence (NIRF) wavelengths (700-1,000 nm) are attractive for potential biomedical applications due to their increased penetration depth and decreased scattering and absorption in tissues relative to the ultraviolet and visible wavelengths [1]. NIRF dyes display strong signal strength, and their chemical modifications can improve the stability of molecular groups and trigger strong fluorescence emission for the use in imaging-based cancer diagnostics [2–4]. Therefore, these dyes represent good molecular probes and are used widely in animal and human imaging studies [5–8]. Indocyanine green (ICG), a representative NIRF dye, has been approved by the US Food and Drug Administration (FDA) for application in medical diagnostics. However, ICG lacks tumour-targeting capability, is not stable, and decomposes rapidly in polar solution. ICG-based tumour imaging often involves non-specific delivery of the dye to tissues other than tumour sites [9–12].

Recently, a group of NIRF heptamethine carbocyanine dyes have been reported to recognize tumour cells directly without chemical modification, suggesting they are promising agents for non-invasive tumour imaging and targeting [13, 14]. Agents such as IR-783 and MHI-148 are heterocyclic polymethine cyanines with dual imaging and tumour-targeting functions [15, 16]. They were shown to be specifically uptaken by cancer cells but not normal cells, as demonstrated in a variety of *in vitro* and *in vivo* models representing different types of cancers [7, 17, 18]. The underlying mechanisms of the dye uptake include tumour hypoxia and the activation of a group of organic anion-transporting peptide (OATP) genes [2, 8].

Hepatocellular carcinoma (HCC) is the fourth most common cancer worldwide [19]. Although a spectrum of methods has been developed for the early detection of different cancer types in order to allow early therapeutic interventions, early detection of HCC is difficult because the symptoms often do not appear until the late developmental stages, leading to a poor survival rate of HCC patients [20, 21]. Additionally, since liver is a major metabolic organ, this represents an additional challenge for the development of molecular probes for HCC imaging. Most agents, if not all, are, to some extent, eventually deposited in the liver, where they release signals, preventing the differentiation of HCC from normal/benign liver tissues, particularly at the early disease stages. Tumour heterogeneity further complicates the information obtained from molecular probes that target a single antigen or metabolic substrate. Therefore, novel agents with increased tumour-targeting specificity for the imaging-based early detection and prognosis of HCC are required.

We investigated the use of heptamethine carbocyanine dye, DZ-1, for the targeting and visualization of HCC, using preclinical models, cell lines, and clinical samples, and explored the mechanisms of dye uptake. DZ-1 fluorescence imaging was applied for intraoperative navigation in a rabbit liver cancer model as well.

## RESULTS

### Specific NIRF dye uptake by liver tumour xenografts

**Figure 1:**
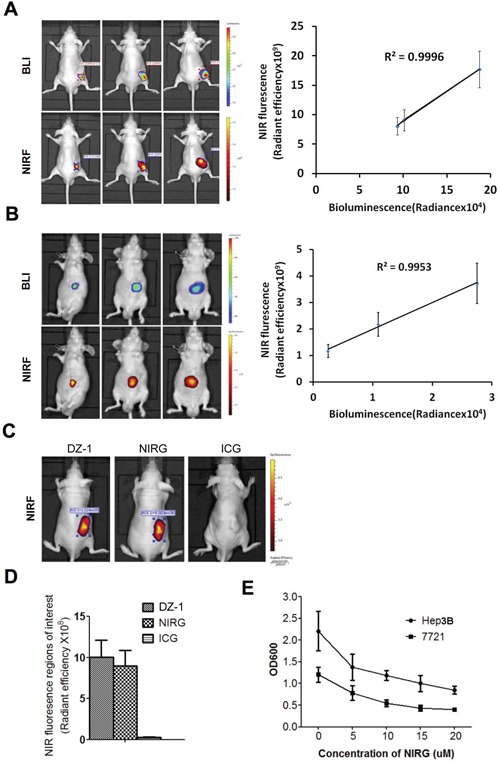
Cellular uptake of dyes in a HCC xenograft mouse model **(A)** NIRF/BLI signal intensity correlation in mice (n=5) with subcutaneous xenografts tumours (right). Representative images are shown (left). **(B)** NIRF/BLI signal intensity correlation in mice (n=5) with orthotopic xenografts tumours (right). Representative images are shown (left). **(C)** Representative images of DZ-1-, NIRG-, or ICG-treated mice subcutaneously xenografted with Hep3B cells. **(D)** Quantification of the uptake of compounds presented in **(C)** by the tumour cells. Data are presented as the signal intensities recorded at the tumour site after subtracting the background signal in a blank region of equal area (mean ± standard deviation (SD), n=5). **(E)** NIRG-treated Hep3B and 7721 growth inhibition curves (mean ± SEM, n=5). *P<0.05, compared with the controls.

To determine NIRF DZ-1 dye uptake by tumour cells *in vivo*, subcutaneous tumour xenografts of Hep3B-Luc cells were established in the nude mice. We determined that both NIRF signals and BLI increased with the tumour growth, and a significant positive association between the levels of these signals was determined (R2=0.9996; Figure 1A). Additionally, we established orthotopic liver transplant model with Hep3B-Luc cells to test the ability of these dyes to detect deep tumours in the hepatic environment. The intensities of both signals at tumour sites were shown to increase steadily as tumours grew, and they showed significant positive correlation (R2=0.9953; Figure 1B).

Furthermore, we compared tumour-targeting capacity of NIRF DZ-1 dyes and a dye-drug derivative, using subcutaneous Hep3B-Luc xenografts. As shown in Figure 1C, DZ-1 and NIRG specifically accumulated in tumours, but not in the normal tissues, while no signals were visualized in ICG-treated mice (Figure 1C and 1D). Moreover, NIRF signals that increased with the tumour growth were detected in NIRG-treated mice (R2=0.99716; Supplementary Figure 1). NIRG treatment significantly inhibited the growth of Hep3B and 7721 cells in a dose-dependent manner, and effective growth inhibition was achieved even at the concentration of 10 mM (Figure 1E), suggesting that gemcitabine antitumor activity was retained in NIRG.

No systemic DZ-1 dye toxicity was observed, and it did not affect mouse body weight. Furthermore, no abnormal histopathologic findings were observed.

### Clinical HCC samples and NIRF dye uptake

**Figure 2:**
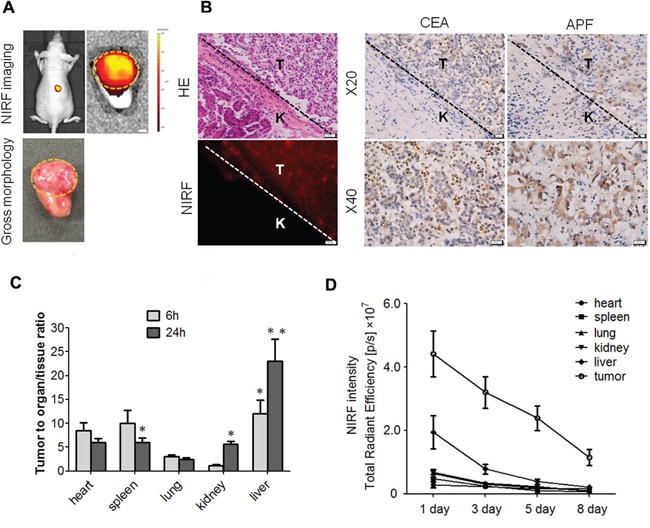
DZ-1 uptake in HCC xenograft and cirrhosis models **(A)** DZ-1 uptake in mice subrenally implanted with clinical liver tumour specimens. *In vivo* (top left) and *ex vivo* approaches (top right). Gross morphology of mouse kidney implanted with human liver tumour tissue (bottom). Representative images are shown. Green dashed circle, tumour area in the kidney. Original magnification: 4×; scale bars, 4 mm. **(B)** NIRF, H&E, and CEA, and AFP analyses of subrenal liver tumour tissues described in **(A).** T and K indicated liver tumor and normal mouse kidney areas respectively, with the tumor-kidney interface specified by dashed lines. Representative images are shown. **(C)** NIRF signal intensities in the tumours and mouse vital organs at 6 and 24 h following the administration of DZ-1 (*ex vivo* imaging). Data are presented as tumour-to-organ ratios. **(D)** NIRF dye uptake in liver tumour and vital organs of mice with Hep3B-Luc orthotopic xenografts during the 8-day observation period (*ex vivo* imaging). Data are presented as tumour-to-organ ratios.

We transplanted fresh tumour tissues obtained from liver cancer patients into the subrenal capsules of nude mice, and examined DZ-1 dye uptake. As presented in Figure 2A, NIRF signals were obtained using both *in vivo* and *ex vivo* fluorescence imaging. We visualized cellular dye uptake in tumour sections under a microscope. We demonstrated that strong intensity of NIRF dye can be observed in the tumour tissues, but not in the normal liver tissues, and the interface between HCC and surrounding normal tissue was clearly defined, as demonstrated by immunohistochemical (IHC) staining performed using two well-established HCC markers, carcinoembryonic antigen (CEA) and alpha-fetoprotein (AFP), which was confirmed by H&E assessment (Figure 2B). Next, we determined the distribution of DZ-1 dye. Two time points, 6 and 24 h after dye injection, were selected to represent short- and long-term exposure to dye. A significantly higher tumour-to-organ ratio was found at both time points when liver was used as a reference organ, compared with the ratios obtained by using other organs for the normalization at 24 h. Sufficiently high ratios were also obtained when using spleen or kidney for the normalisation of NIRF signals at 24 h (Figure 2C). Additionally, a considerably higher retention of NIRF dye was observed in orthotopic tumours compared with that in the normal mouse tissue during the 8-day observation period (Figure 2D).

### Differential NIRF dye uptake in liver cancer and cirrhosis

**Figure 3:**
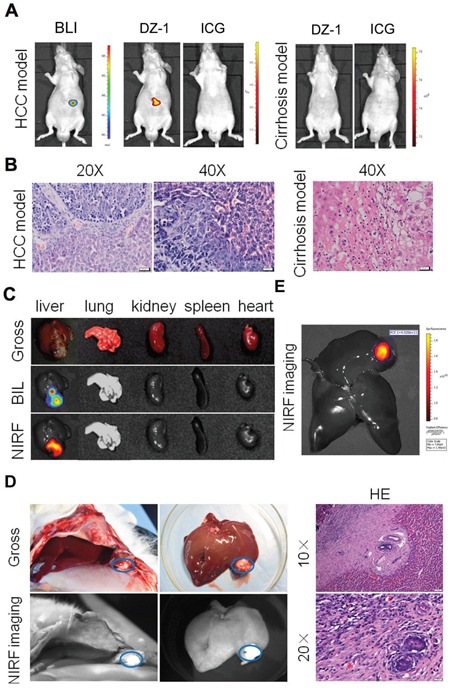
NIRF dye application for the liver cancer detection **(A)** DZ-1 or ICG-treated mice with Hep3B-Luc orthotopic xenografts (left) or cirrhotic liver (right). **(B)** H&E staining of liver tumour and cirrhotic tissues described in **(A).** **(C)** Dual *ex vivo* BLI/NIRF imaging of organs dissected from mice described in **(A).** **(D)** NIRF imaging used for intraoperative guidance during the surgery performed on rabbit VX2 liver cancer (left). H&E staining of the fluorescent tissue sections (right). **(E)** *Ex vivo* NIRF imaging of rabbit VX2 liver cancer.

A critical issue in clinical cancer diagnostics using imaging is a clear differentiation between liver cancer and cirrhosis, which precedes the development of HCC in most cases [22, 23]. Liver cirrhosis in our mouse model was confirmed by H&E staining of liver tissues (Figure 3A and 3B). After DZ-1 treatment of both, cirrhosis and liver tumour groups for 24 h, we detected strong NIRF signals in the liver tumours, in contrast to those in the cirrhotic livers. Quantitative analysis further revealed a six-fold increase in the signal intensity in tumour-bearing mice, in comparison with that in the mice with liver cirrhosis (Supplementary Figure 2A). ICG (10 μmol/kg) was intravenously administered as well, but very weak signals were observed at 24 h in both models (Supplementary Figure 2A). Finally, we excised major organs, including liver, lungs, kidney, spleen, and heart, together with the orthotopic liver tumours, and further imaged them *ex vivo*. A considerably higher fluorescence signal intensity was observed in liver tumours than in other organs (5-20-fold increase; Figure 3C and Supplementary Figure 2B).

### Intraoperative guidance for the determination of liver cancer margins

Intraoperative observation of the liver surface using NIR fibre was performed on all rabbits with VX2 liver tumour. Strong fluorescence signal was detected in the rabbit liver lobes, as seen by gross observation as well (Figure 3D). Liver was analysed further *ex vivo*, and we detected NIRF signal at the same site (Figure 3E). H&E staining of the fluorescent sections showed obvious liver cancer tissue structure, and this was shown to be consistent with the results obtained by surgical exploration and *in vivo* imaging (Figure 3D).

### Cellular uptake of DZ-1 and NIRG

**Figure 4:**
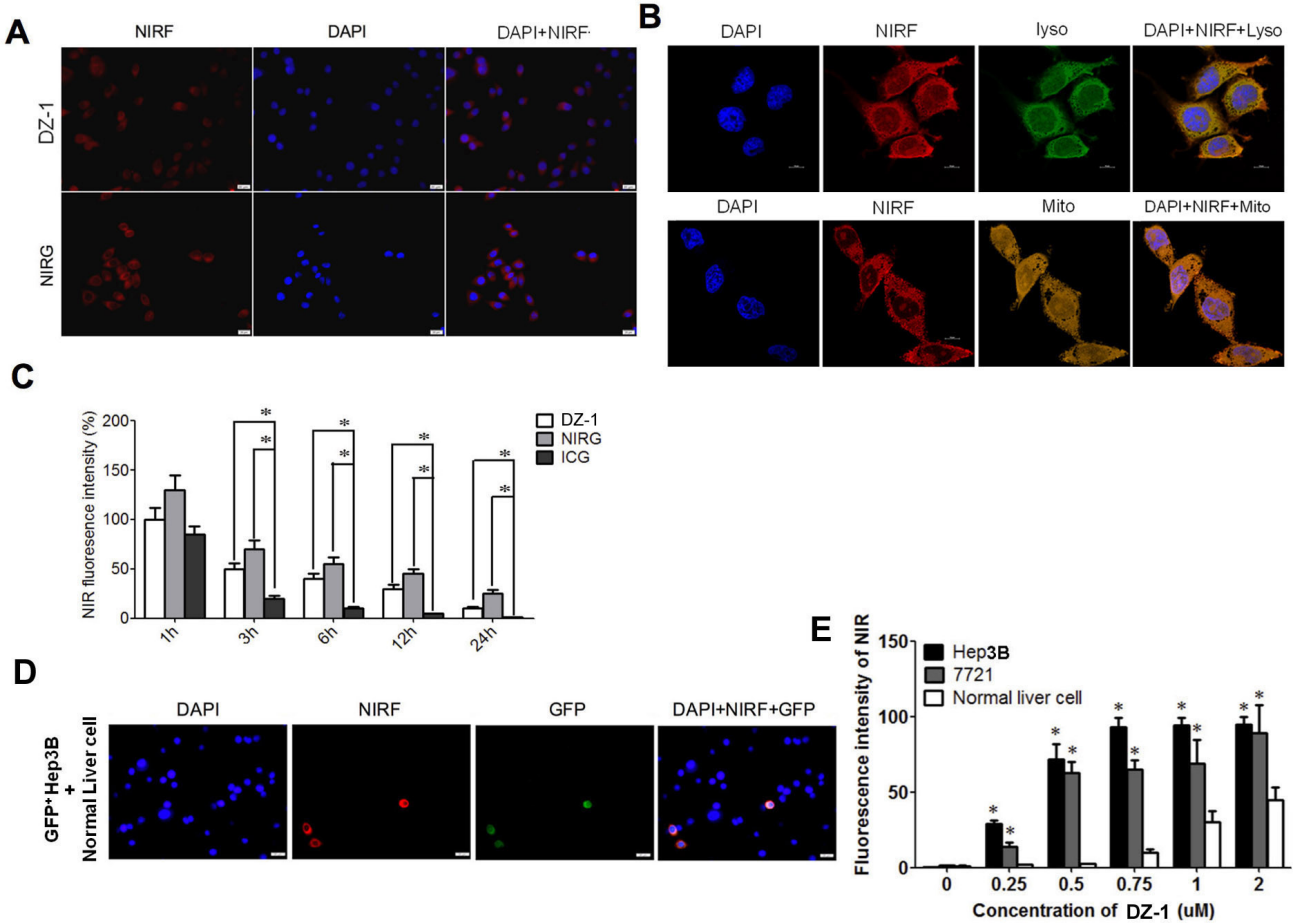
Preferential uptake of DZ-1 by HCC cells **(A)** DZ-1 (top) and NIRG (bottom) uptake by Hep3B cells. Cell nuclei were stained with DAPI. Scale bars, 50 μM. **(B)** Co-localization of DZ-1 (red) and LysoTracker (green, top panel) or MitoTracker (yellow, bottom panel) in Hep3B cells, as determined by confocal microscopy. Cell nuclei were stained with DAPI. Original magnification: 1200×; Scale bars, 10 μm. **(C)** DZ-1, NIRG, and ICG uptake by Hep3B cells at different time points. Signal intensity of DZ-1 at 1 h was considered 100%. *P<0.05. **(D)** Confocal microscope analyses of DZ-1 dye uptake by cells in the co-culture of GFP-labelled Hep3B cells and normal liver epithelial cells. Cell nuclei were stained with DAPI. Original magnification: 400×; Scale bars, 50 μm. **(E)** Flow cytometric analyses of DZ-1 uptake by Hep3B/7721 HCC cells and normal liver epithelial cells (mean ± SEM, n=5). *P<0.05.

We assessed the uptake of DZ-1 and NIRG in HCC cells, and demonstrated, by microscopic examination, that both agents accumulated in Hep3B cells (Figure 4A). To determine the organelle in which NIRF dye preferentially accumulates, we co-incubated Hep3B cells with DZ-1 and NIRG and different organelle-specific trackers. As shown in Figure 4B, DZ-1 was shown to co-localize with MitoTracker (yellow) or LysoTracker (green) in Hep3B cells, indicating the retention of NIRF dye in mitochondria and lysosomes. Additionally, we determined the time-course of HCC cell response to DZ-1 and NIRG treatment, with ICG as a control. All analysed agents displayed significant accumulation in cells, with strong fluorescence signals detected 1 h after the treatment, and this signal was gradually lost during the following 24 h, with the greatest decrease observed in cells treated with ICG, compared with that after the treatment with DZ-1 and NIRG (Figure 4C), which might be due to the faster clearance of ICG than other NIRF dyes.

### Preferential uptake of DZ-1 by HCC cells

To determine whether the tumour-specific uptake of DZ-1 dye observed in tumour xenograft models is recapitulated *in vitro*, we co-cultured GFP-labelled Hep3B cells with normal hepatic epithelial cells. The exclusive uptake of DZ-1 by GFP+ Hep3B cells was observed (Figure 4D). Additionally, we assessed dye uptake by Hep3B and 7721 cells, and normal liver cells, using different concentrations. As shown in Figure 4E, a significantly higher uptake of DZ-1 was observed by the HCC cells, compared with that by normal liver cells at all dye concentrations up to 2 μM, while HCC cells demonstrated a dose-sensitive response at lower DZ-1 doses up to 0.5 μM.

### Hypoxia and OATPs mediate DZ-1 dye uptake by HCC cells

**Figure 5:**
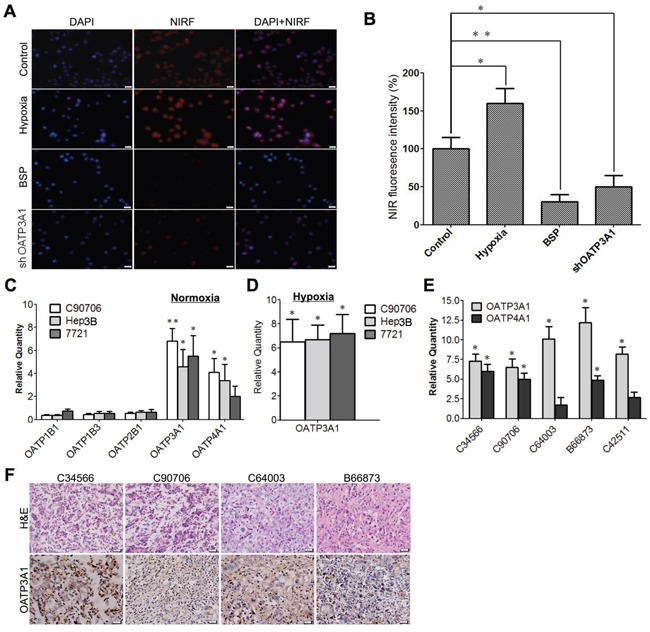
Hypoxia and OATPs mediate NIRF dye DZ-1 uptake in HCC cells **(A)** DZ-1 dye uptake by Hep3B cells with a prior exposure to either hypoxia, BSP, or OATP3A1-targeting siRNA. Representative images are shown. Original magnification: 400×; Scale bar, 50 μm. **(B)** Quantification of DZ-1 dye uptake rate. Signal intensity in the control cells maintained under normoxic conditions was considered 100% (mean ± SEM, n=5). *P<0.05. **(C)** RT-qPCR analysis of OATPs in three HCC cell lines under normoxic conditions. Data are presented as fold changes (mean ±SD) of gene expression levels, compared with that in the normal liver cells. *P<0.05, **P<0.01. **(D)** OATP3A1 expression levels in HCC cells under hypoxic conditions. Data are presented as gene expression fold changes (mean ± SD) compared with those under the normoxic conditions. **(E)** OATP3A1 and OATP4A1 expression in human HCC patient samples. Data are presented as gene expression fold changes (mean ± SD), compared with the adjacent normal liver tissues. **(F)** OATP3A1 expression in samples described in **(E).** Original magnification: 400×; Scale bars, 20 μm.

Hypoxia and OATP genes have been suggested to play important roles in mediating NIRF dye uptake in cancer cells [24]. To explore this, we exposed Hep3B cells to either hypoxic conditions or BSP, a potent OATP inhibitor, which was followed by DZ-1 treatment. As shown in Figure 5A, DZ-1 uptake increased in Hep3B cells under hypoxic conditions but declined in response to OATP inhibition, which was further confirmed by quantitative assessment of fluorescence signal intensity (Figure 5B). We analysed the molecular signature of selected OATP genes that have been reported to have pathologic function in HCC cells [25]. We found that the expression of OATP3A1 and OATP4A1 in Hep3B cells increased, in comparison with that in normal liver cells (Figure 5C). Furthermore, we examined OATP3A1 expression in three different HCC cell lines, one of them, C90706, is a primary cell line derived from a HCC patient sample. We demonstrated that OATP3A1 expression was increased in all cell lines under hypoxic conditions (Figure 5D). To further examine the clinical relevance of OATP3A1 and OATP4A1 genes, we surveyed several HCC clinical samples (C34566, C90706, C64003, B66873, and C42511), which were all shown to overexpress OATP3A1 at mRNA and/or protein levels (Figure 5E and 5F). To investigate whether OATP3A1 expression can directly increase dye uptake, we treated Hep3B cells using OATP3A1-targeting siRNA. Stable knockdown of OATP3A1 expression resulted in a 50% decrease in DZ-1 dye uptake by cancer cells under normoxic conditions (Figure 5A and 5B).

## DISCUSSION

Our group synthesized a series of heptamethine carbocyanine dyes, including IR-783 and MHI-148 [2, 7, 8, 17, 24, 26]. These compounds showed tumour-targeting properties *in vitro* and a nude mouse xenograft model. The sulfonic acid group in the side chain of IR-783 mediates better water solubility and lower toxicity by allowing rapid clearance *in vivo*, but further structural modifications are limited due to the lack of the appropriate chemical functional groups [17]. The side chain carboxylic group of MHI-148 can be conjugated with chemotherapeutics or isotopes, but the molecule is highly hydrophobic [24]. Therefore, we modified MHI-148 structure, in order to improve its water solubility while preserving its chemical manoeuvrability, and one side chain (CH_2_)_5_CO_2_H was replaced with (CH_2_)_4_SO_3_-. The novel compound, designated as DZ-1, has an improved hydrophilicity, so that now the same mode as that used for the clinical ICG administration can be used.

In this study, we demonstrated the ability of a group of NIRF heptamethine carbocyanine dyes to specifically recognize HCC cells and tissue samples in multiple preclinical models. Conventional NIRF dyes can identify tumour cells only after they bind to tumour-specific antigens, peptides, enzyme substrates, and tumour markers [26–30]. In contrast to this, our newly developed heptamethine carbocyanine dyes, including MHI-148 and IR-783, can be taken up directly by tumour cells without chemical binding and time-consuming probe preparation [7, 17]. These dyes have been shown to preferentially accumulate in other types of non-HCC cancer cells without affecting normal cells [2, 17], and *in vivo*, these dyes did not show cytotoxicity or systemic toxicity at a range of doses appropriate for imaging purposes [17, 26].

We demonstrated that a significant correlation exists between NIRF dye uptake by orthotopic liver tumour cells and tumour growth, showing the usefulness of NIRF dye for quantitative assessment of HCC growth and progression. Additionally, NIRF dye-drug conjugate, NIRG, was shown to specifically target liver tumours *in vivo* and effectively inhibit HCC cell growth *in vitro*, suggesting a dual use of NIRG and other similar conjugates as both molecular imaging probes and therapeutic agents for the imaging and treatment of HCC. Furthermore, we demonstrated the superiority of NIRF dyes and their derivatives over ICG for the use in liver tumour imaging. Additionally, NIRF dye was shown to accumulate less in the normal liver than other organs, which facilitates the use of these dyes for HCC imaging specifically, because of the relative reduction in background signal intensity, compared with that observed in other organs.

It has been reported that hypoxia mediates the uptake of NIRF dye by different cancer cells via the activation of HIF1α/OATP signalling [8, 26], which, considering the common molecular aspects shared by different types of cancers, may apply to HCC as well. Here, we showed that the liver tumour-specific accumulation of DZ-1 dye is closely associated with the activation of HIF1α and OATPs, which is consistent with our previous findings [2, 8, 26]. Hypoxic conditions are frequently observed in HCC, with a more robust hypoxic response in the later stages, and have been linked with metabolic changes, neo-vascularization, invasion, metastasis, drug resistance, and, ultimately, poor clinical outcomes [31–33]. This provides a rationale for using NIRF dyes for the visualization and monitoring of HCC progression at different stages.

Additionally, OATPs are involved in the regulation of NIRF dye uptake by HCC cells and tissues. These molecules belong to a group of cell membrane-bound solute carriers, and were originally thought to mediate cellular transport of amphiphilic compounds, including hormones, drugs, and other exogenous substances [34]. Altered OATP expression and different variants were detected in many different cancer types, including HCC [25, 35]. Here, we did not observe an increased expression of OATP1B3 and OATP2B1 in HCC cells, two members in a subfamily of OATPs reported to play a role in the development and progression of prostate cancer and gastric cancer. However, we demonstrated an increase in mRNA expression of other OATPs, including OATP2A1, OATP3A1, and OATP4A1 in HCC clinical samples, compared with that in the normal liver tissue samples [25], while OATP3A1 knockdown in HCC cells was shown to significantly reduce the uptake of NIRF dye. An increased expression of OATP3A1 was demonstrated as well, using the same clinical samples, which represents the first evidence indicating the upregulation of OATP3A1 in HCC clinical samples. Additionally, OATP3A1 was shown to be induced by hypoxic stimuli, suggesting a potential synergistic activity of OATP3A1 and hypoxic signalling in promoting NIRF dye uptake in HCC cells.

NIRF dyes do not have strong inherent ligand-labelling abilities, their signal strength (quantum yield) is weaker than that of the fluorophores of shorter wavelength, and they are generally large molecules that do not penetrate well into tissues [36], since this penetration is highly dependent on charge, size, and solubility of a molecule [37]. NIRF optical imaging agents are unlikely to be used for whole-body non-invasive imaging due to tissue light transport properties. Therefore, fluorescence cholangiography using ICG was mainly developed for the use during the open surgery [38] and laparoscopic surgery [39]. Current clinical application of ICG fluorescence imaging in HCC treatment are limited to the intraoperative navigation [40–42].

ICG is the only NIRF dye approved for medical diagnostic use, but it lacks specificity in the recognition of tumour cells. The uptake of ICG by differentiated HCC cells with concomitant biliary excretion disorder was shown to lead to the accumulation of ICG in cancer tissues [13], which differs from the mechanism of action of heptamethine carbocyanine dyes [43]. A recent study reported that the preferential uptake of ICG by the tumour cells is regulated by the cell membrane-binding ability of ICG, high endocytic activity of tumour cells, and the disruption of tight junctions [44]. This limits the application of ICG for the visualization during surgery. However, heptamethine cyanine dye DZ-1 possesses dual imaging and tumour targeting functions, and it was shown to have significantly higher tumour-targeting specificity, compared with that of the ICG. Using orthotopic liver transplantation rabbit model, we further demonstrated that DZ-1 may be applied for the intraoperative guidance of cancer surgery. As shown in our previous study [44], DZ-1 and ICG concentrations used in the tumour xenograft model were 1 μmol/kg and 10 μmol/kg, respectively. Although the concentration of DZ-1 is one tenth that of IGC, the fluorescence intensity emitted by DZ-1 in the subcutaneous tumour unit area reached 10^9^. When ICG was injected at the same concentration as DZ-1 into the subcutaneous tumour-bearing mice, no difference in NIRF signal intensity between the tumour site and background was observed, indicating that DZ-1 displays a higher fluorescence quantum-yield than ICG.

We did not detect apparent NIRF signals in HCC orthotopic xenograft mice at 24 h after ICG administration, which may be partially due to the rapid decomposition of ICG, resulting in a rapid quenching of ICG fluorescence signals in blood [45]. Although NIRF signals were detected at 1, 3, 6, and 12 h, they were not observed at the tumour sites (data not shown). Unlike that of the DZ-1, ICG uptake by the tumour cells is primarily mediated by passive transport, and therefore, there is no tumour-specific targeting. Additionally, hypoxic conditions may promote the uptake of heptamethine carbocyanine dyes by the tumour cells, but no correlation was observed between ICG uptake rate and the levels of hypoxia in tumour tissue. The results of our study indicate that the levels of hypoxia are crucial for the differentiation between HCC and cirrhotic tissues by DZ-1.

In summary, we successfully applied heptamethine carbocyanine DZ-1 dye for the tumour-specific imaging of HCC *in vitro*, in subcutaneous and orthotopic xenograft mouse models, and using clinical samples. This dye was shown to be applicable as a NIRF imaging probe for the intraoperative guidance during the surgery of rabbit VX2 liver cancer, and it may serve as a platform allowing drug conjugation, in order to achieve both cancer detection and cancer treatment properties. Additionally, we showed that the underlying mechanisms involve the development of hypoxic conditions in tumours and the activation of selected OATP genes that have not been previously reported as relevant for HCC development or treatment, such as OATP3A1. Although the penetration depth of light in tissues is limited for these dyes, we provided novel insights that will allow further development of this group of dyes for the intraoperative guidance during cancer surgery. Therefore, the results of this study may lead to the clinical development of this dye in the future.

## MATERIALS AND METHODS

### Cell lines and reagents

**Figure 6:**
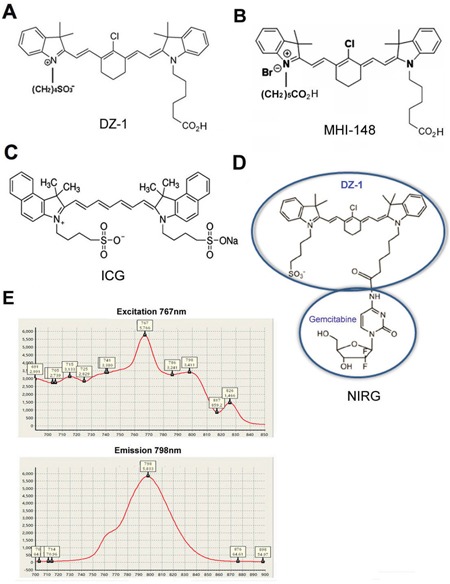
Chemical structures and spectral characteristics of heptamethine carbocyanine dye **(A-D)** Chemical structures of DZ-1, MHI-148, ICG, and NIRG. **(E)** Spectral characteristics of DZ-1.

Human HCC Hep3B2.1-7 (Hep3B; Typical Culture Preservation Commission Cell Bank, Chinese Academy of Sciences, originally provided by ATCC, USA) and SMMC-7721 (7721; kindly provided by Prof. Jiianli Jiang, FMMU, Xi’an, China) cell lines were used [46]. Primary HCC cell line, C90706, was derived from a clinical HCC patient specimen. Lentiviral particles expressing both luciferase (Luc) and green fluorescence protein (GFP) were purchased from GenTarget (San Diego, CA, USA). Luc- and GFP-tagged Hep3B cells and normal human hepatic epithelial cells were prepared following routine lentiviral infection procedures. These cells were cultured in Minimum Essential Medium (MEM) supplemented with 10% foetal bovine serum (FBS) and 1% penicillin/streptomycin (Thermo Scientific, Waltham, MA, USA). Cell line authentication was performed by short tandem repeat (STR) profiling as LGC Standards. In order to induce hypoxia, cells were grown in a hypoxic chamber (1% O_2_, 5% CO_2_). ICG dye and bromosulphophthalein (BSP), an OATP inhibitor, were purchased from Sigma-Aldrich (St. Louis, MO, USA). OATP3A1-targeting short hairpin (sh)RNA-carrying lentiviral particles, used for the stable knockdown of these genes, were purchased from Santa Cruz (Santa Cruz, CA, USA). DAPI was purchased from Tiangen (Shanghai, China). Heptamethine carbocyanine dyes, DZ-1 and DZ-1 analogue-gemcitabine conjugate (NIRG) were kindly provided by Dr. Leland W.K. Chung (Cedars-Sinai Medical Center, Los Angeles, CA, USA) [2, 17]. The chemical structures of these compounds are presented in Figures 6A-6D. DZ-1 emission spectrum in phosphate-buffered saline (PBS) was detected by a spectrophotometer (Figure 6E). Optimal excitation wavelength of DZ-1 was 767 nm by full spectrum excitation, while emission wavelength was 798 nm.

### Animals

Six-seven-week-old male BALB/c nude mice were purchased from Vital River (Beijing, China) and housed in a pathogen-free system at the Laboratory Animal Center of the Fourth Military Medical University (FMMU, Xi’an, China). Mice were anesthetized with ketamine (100 mg/mL, i.p.) and xylazine (20 mg/mL, i.p.) and maintained under isoflurane during surgery and imaging. New Zealand white rabbits (2.5-3 kg) were fed with standard chow and water, and housed in the animal facility. Rabbit VX2 liver tumours were induced by injecting tumour homogenate into the thigh muscle of a rabbit. All animal experiments were approved by the Institutional Animal Care and Use Committee of FMMU (protocol No. 16013). All methods were carried out in accordance with the approved regulations of FMMU.

### Clinical specimens

All fresh liver specimens of cancer patients were obtained from the Xijing Hospital of the FMMU, and informed consents were obtained from all patients. The use of these specimens in research was approved by the medical ethics committee of the Xijing Hospital. All methods were performed in accordance with the approved guidelines.

### NIRF imaging of tumour xenografts in mice

Hep3B-Luc cells (1×10^6^, 3×10^6^, and 9×10^6^) were either inoculated subcutaneously into the right flank of the nude mice or subjected to orthotopic liver transplantation (OLT) to the nude mice according to the following protocol. Briefly, a 100-μL mixture of ketamine and xylazine was intraperitoneally injected into mice. A small cut from the medioventral line with ophthalmic scissors enabled full exposure of the liver, and Hep3B-Luc cells in 50-μL volume were injected into the porta hepatis. After returning the liver to the abdominal cavity, abdominal muscles and skin were sutured. Two weeks later, the operated mice were intravenously injected with DZ-1/NIRG (1 μmol/kg) or ICG (10 μmol/kg) [44]. NIRF intensity in the regions of interest was measured using a Lumina II Small Animal Optical Imaging System (Caliper Life Sciences, Hopkinton, MA, USA) 24 h after dye administration. In parallel, luciferase substrate (Gold Biotechnology, Olivette, MO, USA) was intraperitoneally injected into mice at the dose of 1.5 mg/mouse, and bioluminescence intensity (BLI) at the tumour sites was measured using the same imaging system. We assessed the correlation between NIRF signal intensity and BLI.

### NIRF imaging for the intraoperative guidance during rabbit VX2 liver cancer surgery

Rabbits were anesthetized with intravenous ketamine (10 mg/kg) and xylazine (3 mg/kg). Under aseptic condition, a mini-laparotomy was performed in the subxiphoid area, exposing the liver. An incision of 1 cm was made in the liver lobe. Two small rabbit VX2 tumour pieces of 3 mm^3^ each were implanted into the hepatic incision. Pressure was applied over the puncture site using gelatine sponge for approximately 2 min. Abdominal muscles and skin were sutured [47]. Two weeks after tumour implantation, the rabbits were intravenously injected NIRF dye DZ-1 (1 μmol/kg). Following the dye administration, 12 h after that, anesthetized rabbits were celiotomised to expose liver. Liver tumour margins were detected by NIRF optical fibre exploration instrument (Chinese Academy of Science, Institute of Automation, Device ID. 140800000099004) [48]. The removed the liver was washed with PBS and optical imaging was performed as previously described. Tissue was fixed using 4% formalin for the histopathologic analysis.

### DZ-1 uptake by HCC cells

To determine the specific uptake of DZ-1 by HCC cells, GFP-tagged Hep3B cells were co-cultured with normal human hepatic epithelial cells at a ratio of 1:10. Cells were seeded in petri dishes, and DZ-1 (20 μM) was added 24 h after the seeding. Cells were incubated at 37°C for 30 min, followed by PBS wash. Dye uptake was analysed using a NIRF microscope equipped with an ICG filter (excitation/emission: 750-800/820-860 nm). Cell nuclei were stained with DAPI. To determine the organelle-specific cellular localization of NIRF dye, Hep3B cells were seeded on petri dishes, exposed to MitoTracker (200 nM) or LysoTracker (75 nM) 24 h after seeding, and incubated at 37°C for 30 min. DZ-1 (20 μM) was added, and another 30-min incubation was performed at 37°C, followed by the PBS wash. The uptake of probes and dyes was determined by microscopic examination as described. To determine the effects of hypoxia and OATP inhibition on DZ-1 uptake, Hep3B cells were seeded and treated with DZ-1 (20 μM) 24 h after the seeding. Cells were cultured for 24 h under 1% O_2_ hypoxic conditions or with a prior exposure to BSP (250 μM, 1 h). Cells were washed with PBS, and cell nuclei were stained with DAPI after fixation. Control groups were maintained in normoxic conditions or treated with a vehicle.

### Liver cirrhosis model

We established a carbon tetrachloride (CCI_4_)-induced cirrhosis mouse model [49]. Briefly, CCl_4_ was dissolved in olive oil (1:5), and 1 μL/μg body weight of CCl_4_ was injected intraperitoneally into 5-week-old male nude mice twice weekly for 5 weeks. In parallel, orthotopic liver tumour xenografts were established using Hep3B-Luc cells. DZ-1 (1 μmol/kg) was intravenously injected into liver tumour and liver cirrhosis mice. Whole-body fluorescence imaging was used to determine the differential uptake of this dye.

### Reverse transcription and quantitative PCR

Total RNA from fresh tumour tissues or cultured cells was isolated using RNAsimple Total RNA Kit (Tiangen, Beijing, China) and reverse-transcribed to cDNA using the ReverTra Ace qPCR RT Kit (Takara, Dalian, China). The expression levels of the OTAP gene family members were determined. PCR analysis was performed using the StepOnePlus Real-Time PCR System (Thermo Fisher Scientific). All primer sequences used are listed in Supplementary Table 1.

### DZ-1 toxicity *in vivo*

We investigated the toxicity of DZ-1 in by intravenously injecting the dye. The BALB/c mice (n=10 per group) were divided into four groups. The control group received PBS, while the treatment groups received daily DZ-1 intravenous injections, at the following doses: 1 μmol/kg (imaging dose), 50 μmol/kg, and 100 μmol/kg. The mice were weighed daily, and their physical activities were observed for 1 month after the dye injection. The histopathological morphology of their vital organs, including heart, liver, spleen, lungs, and kidneys were assessed at the time of sacrifice.

### Statistical analysis

All values in figures are presented as mean ± standard error of mean (SEM) obtained in at least three independent experiments. Comparisons between the Kaplan-Meier curves were performed using the long-rank test. All other comparisons were performed by using unpaired 2-tailed Student's t test. P≤0.05 was considered significant.

## SUPPLEMENTARY MATERIALS FIGURES AND TABLE


