# Supplementary material for: Heptamethine carbocyanine DZ-1 dye for near-infrared fluorescence imaging of hepatocellular carcinoma
Source: Oncotarget. 2017 May 24;8(34):56880–92. doi: 10.18632/oncotarget.18131 (PMC5593610; doi:10.18632/oncotarget.18131)
Supplement: Supplementary file 1 [file oncotarget-08-56880-s001.pdf]

Heptamethine carbocyanine DZ-1 dye for near-infrared fluorescence imaging of hepatocellular carcinoma

SUPPLEMENTARY MATERIALS

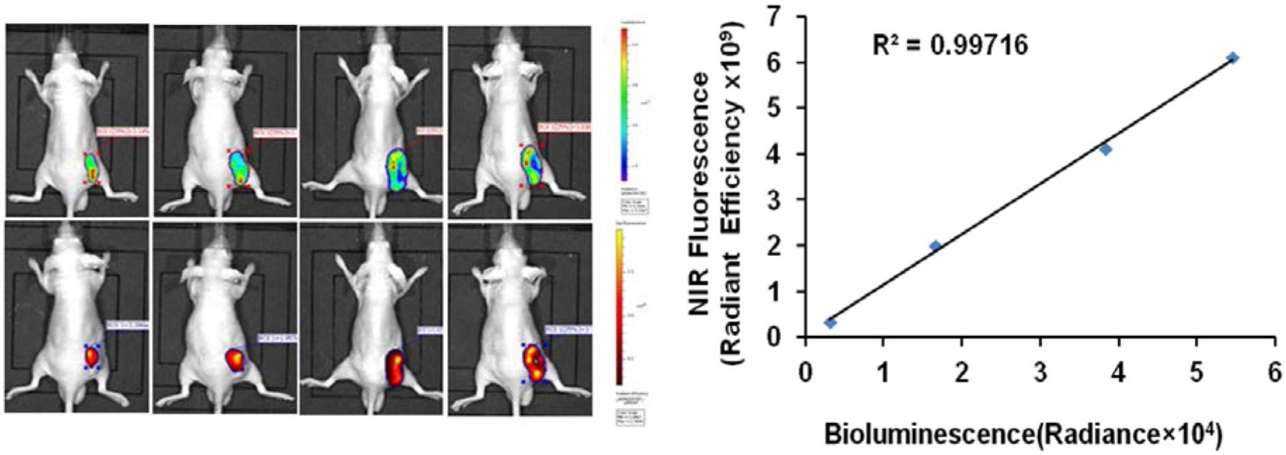

Supplementary Figure 1: Quantitative assessment of tumor growth by NIRG-mediated fluorescence imaging. Mice bearing subcutaneous Hep3B-Luc xenografts were administered with NIRG followed by dual NIRF/BLI imaging. Images of respresentative mice are shown **(A)**. Quantitative correlation between tumor NIRF and BLI signal interest was established (n=5) **(B)**.

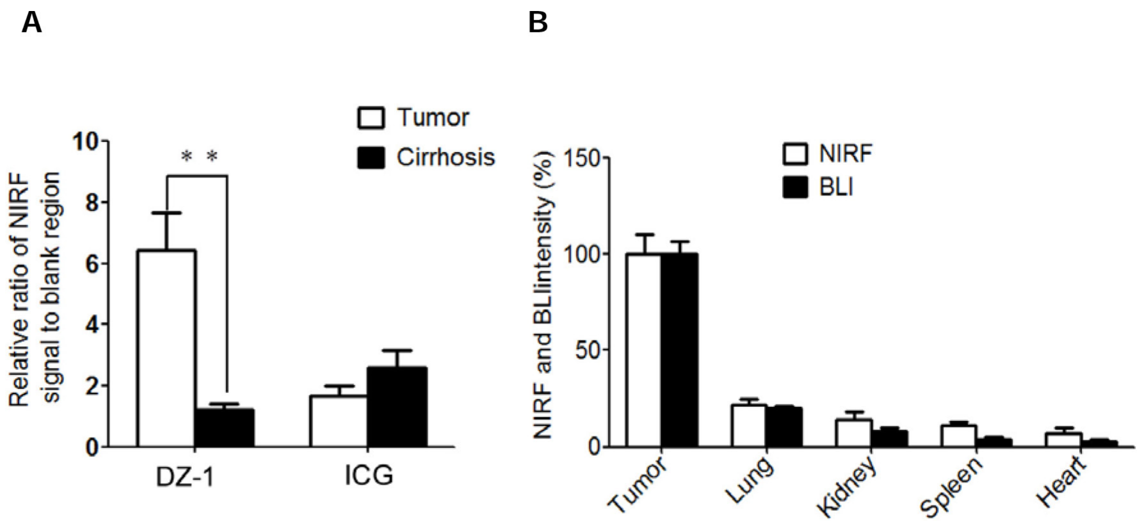

Supplementary Figure 2: Quantification of tumor NIRF/BLI signals in HCC xenograft and cirrhotic models. **(A)** Quantification of NIRF signals in mice receiving either DZ-1 or ICG for both Hep3B orthotopic xenograft and chemically induced cirrhotic models as described in Figure 3A. Data are presented as NIRF signal intensity at tumor site after subtraction of background signal intensity in a blank region of equal area (mean ± SD, n=5). **$p<0.01$. **(B)** Quantification of NIRF/BLI signal intensity in liver tumor and select major organs dissected from mice as described in Figure 3C (mean ± SD, n=5). Signal intensity in liver tumor was set as 100% for both NIRF and BLI groups.

Supplementary Table 1: Primer sequences of OATP gene family used for RT-qPCR

OATP1B1	F: GTCACCATCCTGGAGCTGTT
	R: GAAGGCCGTGTTGACGATAC
OATP1B3	F: GGGTGAATGCCCAAGAGATA
	R: ATTGACTGGAAACCCATTGC
OATP2A1	F: GTGGTGAACCAGGAGGAAAAG
	R: GTATAGGCAGGTGTGGAAGAG
OATP2B1	F: TCAAGCTGTTCGTTCTGTGC
	R: GTGTTCCCCACCTCGTTGAA
OATP3A1	F: TGAGCCAGTCTGTGGATCAG
	R: ATCACTTGGCGACTTTGGAC
OATP4A1	F: CTGCCAGCCAGAACACTACA
	R: AGAAGGAGGGGCTTTCTCTG
OATP5A1	F: GACAACAGCCTCAAGATCATCAG
	R: ATGGCATGGACTGTGGTCATGAG
GAPDH	F: GACAACAGCCTCAAGATCATCAG
	R: ATGGCATGGACTGTGGTCATGAG
